# Spatial variations in the stable isotope composition of the benthic algae, *Halimeda tuna*, and implications for paleothermometry

**DOI:** 10.1038/s41598-020-73283-x

**Published:** 2020-10-01

**Authors:** M. Dale Stokes, James J. Leichter, Stephen R. Wing

**Affiliations:** 1 University of California San Diego, Scripps Institution of Oceanography, La Jolla, CA 92039 USA; 2 Department of Marine Science, University of Otago, PO Box 56, Dunedin, 9054 New Zealand

**Keywords:** Palaeontology, Marine biology, Palaeoecology

## Abstract

On Conch Reef, Florida Keys, USA we examined the effects of reef hydrography and topography on the patterns of stable isotope values (δ^18^O and δ^13^C) in the benthic green alga, *Halimeda tuna*. During the summer, benthic temperatures show high-frequency fluctuations (2 to 8 °C) associated with internal waves that advected cool, nutrient-rich water across the reef. The interaction between local water flow and reef morphology resulted in a highly heterogenous physical environment even within isobaths that likely influenced the growth regime of *H. tuna*. Variability in *H. tuna* isotopic values even among closely located individuals suggest biological responses to the observed environmental heterogeneity. Although isotopic composition of reef carbonate material can be used to reconstruct past temperatures (T(°C) = 14.2–3.6 (δ^18^O_*Halimeda *_− δ^18^O_seawater_); r^2^ = 0.92), comparing the temperatures measured across the reef with that predicted by an isotopic thermometer suggests complex interactions between the environment and *Halimeda* carbonate formation at temporal and spatial scales not normally considered in mixed sediment samples. The divergence in estimated range between measured and predicted temperatures demonstrates the existence of species- and location-specific isotopic relationships with physical and environmental factors that should be considered in contemporary as well as ancient reef settings.

## Introduction

Calcareous macroalgae of genus *Halimeda* (Chlorophyta, Bryopsidales) are important benthic autotrophs in subtropical and tropical waters worldwide^1–3^ where they can be found from the shallow subtidal to depths exceeding 100 m^1,4–6^. In addition to being important primary producers in many ecosystems, they are an import aragonitic sediment source via reproduction, physical fragmentation, and herbivory^7–10^. In some reef settings, calcareous algae can contribute more than 60% of the total carbonate deposition^11^ and in the Florida Keys, species of *Halimeda* can contribute more reef sediment than either coral or coralline algae^12^. Because of the conspicuous sedimentary particles made by *Halimeda* sp., they have produced an important paleontological record since the early Miocene^13–15^ and have proven useful for environmental reconstructions and biostratigraphy^16–21^.

The stable isotopic composition of sedimentary fragments composed from calcifying organisms, like *Halimeda*, can provide information regarding the integrated chemical and physical environmental conditions of the surrounding seawater during precipitation following the foundational work of Urey and colleagues^22–25^. The analysis of variations in δ^18^O, particularly when integrated with additional data from elemental ratios such as strontium:calcium and barium:calcium, have been used to imply natal origin in pelagic larvae^26^, infer variability in marine salinity^27^ as well as estimate oceanographic paleo temperature time series^28^. The degree to which the carbonate isotopic values are in equilibrium with the surrounding seawater depends upon the physical thermodynamic kinetics during calcification—diffusion, hydration and hydroxylation of CO_2_—and the degree of metabolically driven fractionation associated with general physiology, respiration and photosynthesis (often termed the ‘vital effects’). Ultimately, the preserved isotopic signature depends upon both these exogenic and endogenic kinetic factors, the distribution of isotopes in the *Halimeda* fragments, the contribution of *Halimeda* to the total sediment record, and any syndepositional and later diagenetic effects^29,30^.

An individual *Halimeda* consists of a holdfast comprised of unorganized coenocytic filaments, and a series of branching segments with lengthwise oriented filaments and a laterally displaced cortex with a semi-isolated intercellular space^31^. The crystallization of aragonite is mediated by photosynthetic uptake of CO_2_ (see review in^32,33^) and occurs in three stages in the intercellular space. There is first the growth of fine-grained aragonite into the filament walls, followed by the growth of aragonite needles perpendicular to the filaments and into the intercellular space. The final step involves the crystallization of any remaining space with irregularly arranged aragonite needles^16,35,36^. Most evidence suggests that oxygen isotopes in *Halimeda* aragonite are precipitated in thermal isotopic equilibrium with the surrounding seawater^16,32,34,37,38^. Because the resulting ^18^O isotopic value is determined by physical environmental conditions (i.e. temperature) during calcification, it is believed that *Halimeda*, under favourable circumstances, can be used to reconstruct seawater temperature in shallow-water environments analogously to the widespread use of benthic foraminifera from deep sea sedimentary environments^37^.

In contrast to stable oxygen, metabolic processes affect isotopic fractionation and the incorporation of CO_2_ into biogenic pathways that influence δ^13^C values of *Halimeda* aragonite^39^. Fractionation of carbon reservoirs associated with photosynthesis and respiration as well as during calcification lead to variable disequilibrium in δ^13^C values depending upon light level, growth stage and environmental conditions which can vary substantially in both time and space in a reef ecosystem^16,32,35–41^. Recent research has also examined the varying and differential effects of temperature, pH and light intensity on photosynthetic rates and calcification in *Halimeda* spp. in light of ocean acidification and warming^36,42–49^_,_ although their coincident biogenic effects on the isotopic carbon signature has only received limited study to date^46,50,51^.

**Figure 1:**
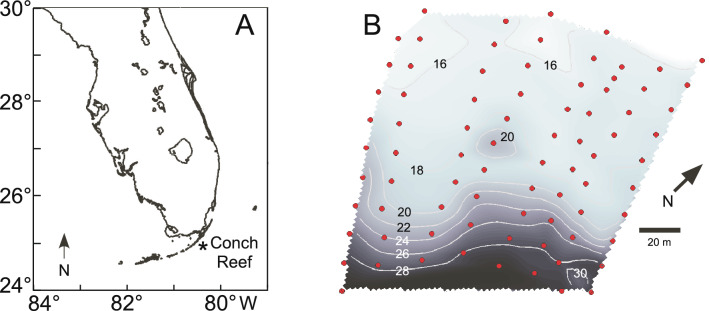
Site Location. (**A**) Map of the study site at Conch Reef (24°57.0′ N, 80°27.3′ W) Florida, U.S.A. Map created using MATLAB (R2018a)^102^. (**B**) Detailed bathymetry, and Benthic Oceanographic Array (BOA) sensor node locations at the study site. Sensor locations sampling sites are indicated by the red dots. The depths of selected contour intervals are indicated by the numerals in 2 m increments and shown as the grey lines over the grey scale background (deeper = darker).

In the present study we describe temporal and spatial variability in both seawater temperature and in *Halimeda tuna* isotopic composition sampled over the irregular surface of a coral reef in the Florida Keys. The site chosen was Conch Reef, within the Florida Keys Marine Sanctuary, which has been the location of several decades of detailed study associated with NOAA and the National Undersea Research Center (Fig. 1). Details of the fine-scale spatial and temporal variability in the physical oceanography at this site can be found in Leichter et al.^52,53^ who deployed a dense array of (100 +) thermistors along the reef from June through September in 2003 and 2004, as well as on a less dense spatial scale almost continuously from 1992 to 2010^53–55^. These studies have shown that during approximately half the year, May through September, the water column and near bottom hydrography of Conch Reef is strongly influenced by the episodic incursion of cool, sub-thermocline water from offshore of the reef slope, driven by strong semidiurnal internal tides and higher frequency internal wave activity (see also^56^). The offshore water masses interact with the reef bathymetry producing a temporally and spatially heterogeneous temperature and nutrient environment both across and within reef isobaths. Most importantly, the water flux creates areas with persistent warm and cool temperature anomalies relative to mean thermal conditions, that are closely associated with the heterogeneous reef topography. The pooling of cool, dense water constrained within pockets and depressions of low relief can remain for several hours following individual incursion events, and the events typically occur up to twice per day. This implies that there could be spatially distinct geochemical signals of the thermal anomalies detected in variability of isotopic values in calcifying benthic algae. This further implies that it may be important to recognize the sources and magnitude of inherent spatial variability when attempting to interpret patters of isotopic values through time, for example in the case of reconstructing past temperature patterns from historical to ancient reef sediments.

The cool water intrusions are also associated with increased levels of nutrients and greater plankton densities, up to an order of magnitude higher than background levels, along with temperature fluctuations as great as 8 °C within minutes with durations of multiple hours^53,57^, and high frequency upwelling appears to be a significant overall nutrient source for the outer reef slopes^53,61,62^. Sampling of the water column seaward of Conch reef, indicated that cooler offshore water had a nearly linear increase in total inorganic nitrogen with decreasing temperature^54^ while at specific locations on the reef, integrated cooling degree-hours below 26 °C could be used as an index of net nutrient exposure and was strongly correlated with tissue δ^15^N in benthic macroalgae^51^. During the summer, the thermocline seaward of Conch reef was often relatively shallow (50 m depth), and upward incursions of cool (22–26 °C) water onto reef slopes occured frequently. The relatively high concentrations of subsurface nitrate (5–20 μmol L^−1^) and soluble reactive phosphate (0.1–2.0 μmol L^−1^) below the thermocline^53,58^ indicated the potential for the offshore nutrient pool to be an important source of nutrients reaching the reef slope, especially at depths of > 10 m as incoming internal wave bores mixed subthermocline water with warm surface waters.

During the winter months, the water temperatures at Conch Reef can be on average up to 8 °C colder than the summer maximum temperatures and occasional atmospheric cold fronts can cause surface temperatures to occasionally drop below 20 °C for short periods (i.e. order one to several days). Cooling during the fall and winter (October through January) was associated with the breakdown of water column stratification and a reduction in subsurface thermal variability due to internal waves. The thermal structure and current flow patterns impacting Conch Reef are thus compounded by the localized seasonal variability in stratification that modulates high-frequency variation associated with internal wave production^55–60^.

Here we describe spatial patterns in the stable isotopic signature of *Halimeda tuna*, collected at multiple locations across Conch Reef, for comparison to measured patterns in benthic temperature that resulted from interactions of local hydrography and bathymetry. Because we compared the spatial distribution of the isotopic signatures and benthic temperature parameters as 3-dimensional maps of the landscape, rather than as a time series from a single spatial location, we used a geospatial analysis technique in order to quantify the similarity in the landscape maps. This provided more details on potential sources of heterogeneity in contemporary carbonate biogeochemistry that differentially affect isotopic values of δ^18^O and δ^13^C from sessile autotrophs, like *Halimeda*, that improve our understanding of the importance of integrating environmental variation and spatial heterogeneity into analysis of paleocarbonates and modelled paleoclimate indicators. We also used the δ^18^O values to compare models of the predicted seawater temperature during *H. tuna* carbonate formation to the observed spatial and temporal variation of benthic water temperature across the study site. The enhanced resolution of variability in both isotopic values and environmental conditions allows for a more accurate calibration for δ^18^O-based paleothermometers using *Halimeda* for new, or existing, paleorecords in the Florida Keys or other locations with highly variable physical environments.

## Results

### Temperature variability

**Figure 2:**
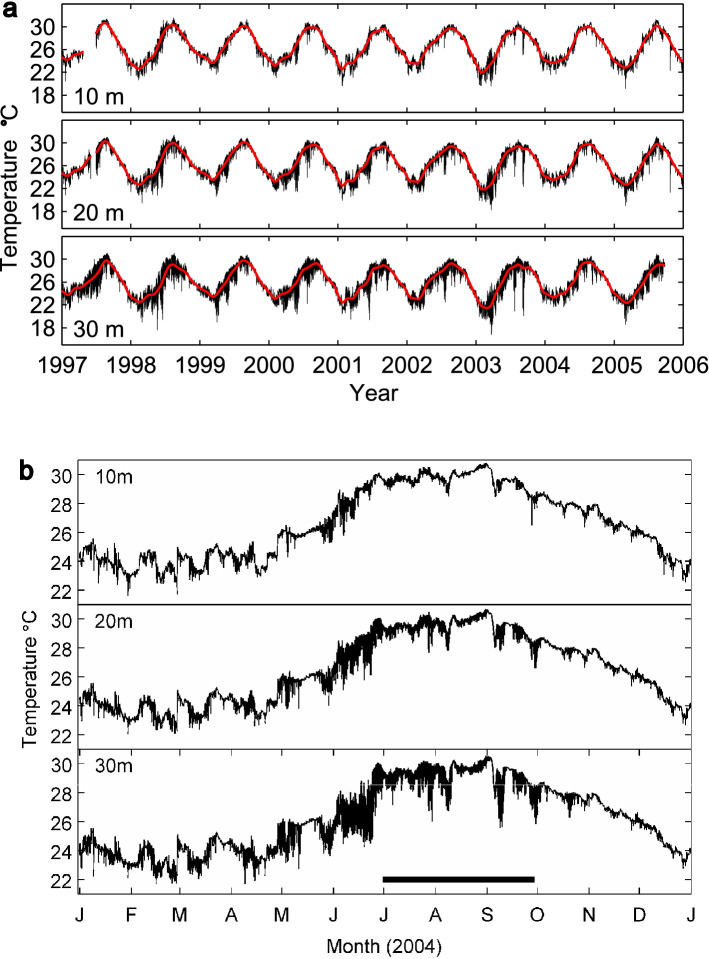
Temperature variation on Conch Reef. Variation in water temperature at Conch Reef recorded at the 10, 20 and 30 m isobaths. Data were sampled at 2 min intervals and averaged in 10 min bins for plotting the complete data set. (**A**) Data collected from 1997 through 2006. Red line indicates the average temperature. (**B**) Expanded view of water temperature during 2004 show high temperature variability. The period of high-resolution data collection from the Benthic Oceanographic Array, during July through September is indicated by the black horizontal bar.

**Figure 3:**
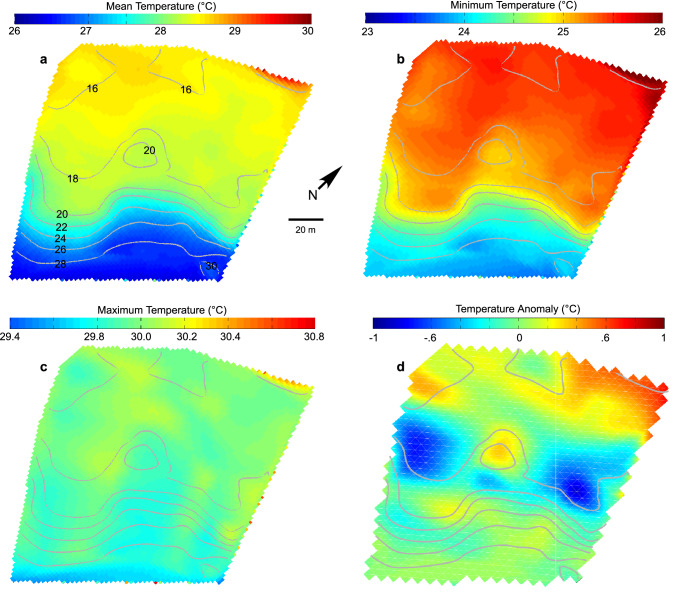
Spatial variation in benthic temperature. Temperatures recorded by the Benthic Oceanographic Array over the sample area at Conch Reef during the summer (June–September) 2004 and interpolated onto the reef bathymetry. Details of BOA temperature analysis can be found in^52,91^. Depth contours, scale and orientation are the same for all subfigures. Note that the temperature scale varies between subfigures to highlight the differences. (**A**) Mean temperature. Numerals on grey contour lines indicate depth in m. (**B**) Minimum temperature. (**C**) Maximum temperature. (**D**) Temperature anomaly along isobaths.

The benthic water temperature on Conch Reef varies about 8 °C with season and depth (Fig. 2). The yearly mean temperature and standard deviation at 10, 20 and 30 m depth, corresponding to the shallow, the mid, and base of the fore reef slope, is 26.60 ± 2.43 °C, 26.49 ± 2.38 °C, and 26.22 ± 2.35 °C respectively. The yearly minimum and maximum temperatures from the long-time series sensors, are similar across the 10, 20 and 30 m depths due to periods of intense mixing, however the summary data averaged from a single sensor do not adequately describe the large spatial and temporal heterogeneity in the temperature field along the reef. The minimum and maximum temperature was 21.60 °C and 30.81 °C at 10 m depth, 22.04 °C and 30.72 °C at 20 m depth, and 21.67 and 30.54 °C at 30 m depth. During the summer months the water column seaward of the reef is strongly stratified. Superimposed on the general seasonal warming of the surface layers, is extensive high frequency variability associated with incursions of cool, deep water up the reef slope (Fig. 2B). These temperature fluctuations are driven by the repeated arrival of internal waves and can be greater than 8 °C on a time scale of minutes to tens of minutes with a concentration in variability at the M2 tidal frequency^53,57^. Temperature variability is greatest at the base of the reef slope and the deeper depths are associated with the lowest mean temperatures because not all cold incursions advect all the way up the reef slope (Fig. 3A–C). The coldest minimum temperatures are also found at the deepest depths, however, it should be noted that there are periods when the entire water column, from the 30 m isobath to the surface is isothermal and may exceed 30 °C (i.e. during August and September). As previously reported^53,54,57^, density stratification of the water column seaward of the reef is primarily driven by temperature changes (e.g. summer time surface temperatures 28–30 °C, and temperatures at ~ 100 m depth below the thermocline is 12–14 °C). There also can be slight water column variation in salinity, between 35 and 36.5 PSU in near-surface waters less than 100 m deep. Salinity time series measured on the reef slope at 21 m, can sometimes show both increases and decreases in salinity on order 0.1 to 1 PSU on the same time scale as the large fluctuations in temperature associated with internal waves^53,54^ (and Leichter unpublished data). However, the small density differences due to salinity variations are not as important as the large density changes due to temperature variation that then control the density-driven and topographically-constrained flow of water across the benthos^57^.

As shown by the temperature anomalies in Fig. 3C, there are variations in benthic temperature that are independent of depth that are a consequence of the reef topography. Non-zero values of the horizontal mean temperature anomaly imply site-specific deviations from the mean profile of temperature at a given depth. For example, the anomalous temperatures along the 18–20 m isobath are associated with variations in temperature in and around a 2–3 m depression in the reef where dense, cold water that has been advected up-reef pools and collects rather than immediately flowing back down slope. The reef deeper than about 22 m in this location shows little horizontal variation in temperature along isobaths.

### δ^18^O and δ^13^C isotopic distribution

There were no significant differences in the seawater δ^18^O values across depths from 3 to 80 m as determined by ANOVA (F(3,22) = 1.54, p = 0.24). The mean and S.D. of δ^18^O was 0.93 ± 0.05, 0.97 ± 0.06, 0.93 ± 0.05, 0.96 ± 0.15, 0.86 ± 0.17, for the 3, 40, 65 and 80 m samples respectively. This result is similar to the reported seawater δ^18^O of + 1.0 estimated for this region from the Global Seawater Database^63^. There was a significant difference in the water collected from approximately 100 m depth with a mean and S.D. of 0.63 ± 0.16 using a Tukey HSD post hoc test.

**Figure 4:**
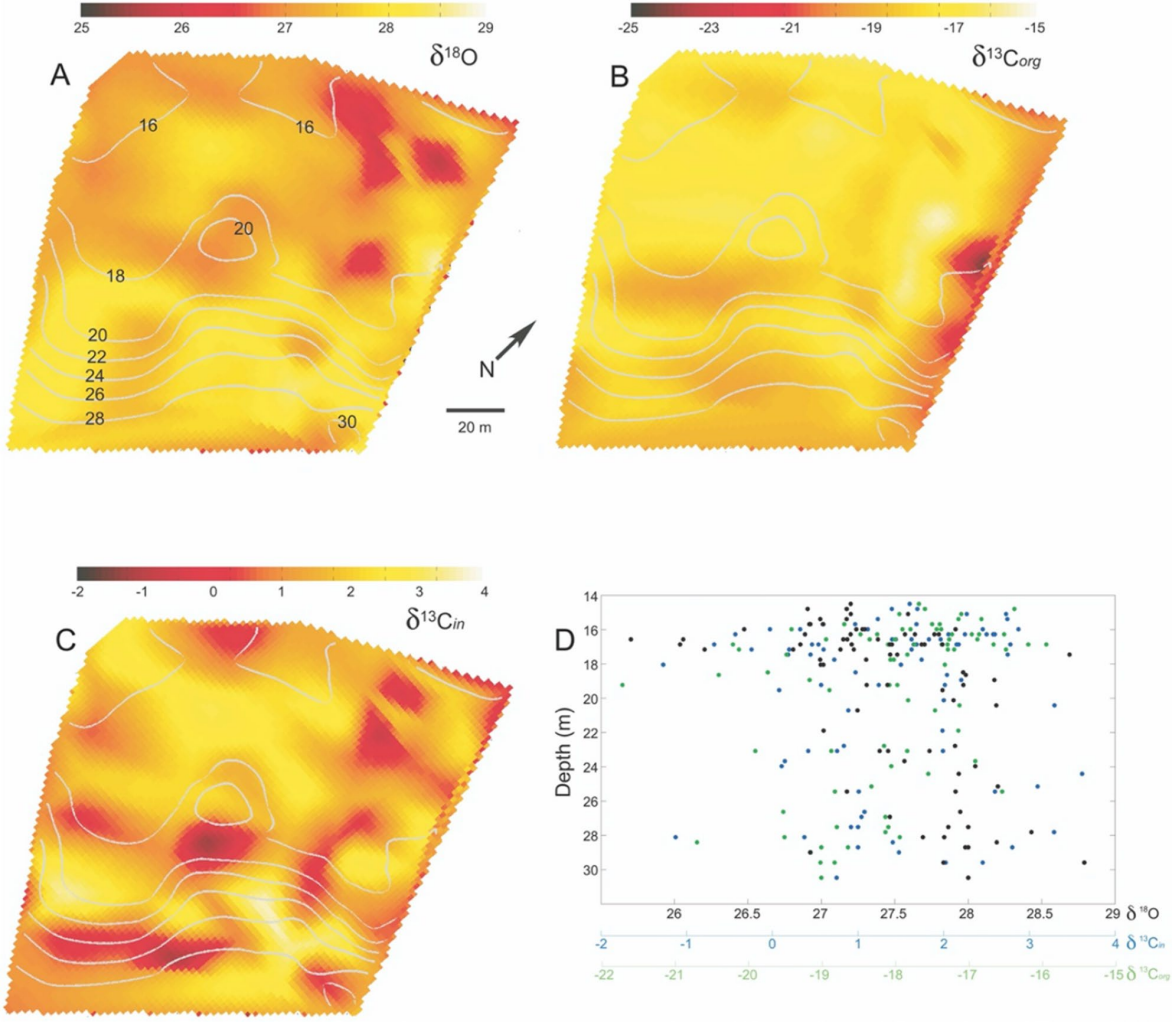
*Halimeda tuna* δ^18^O and δ^13^C*.* Isotopic analysis from *Halimeda tuna* samples collected at the Benthic Oceanographic Array node locations (see Fig. 1) and interpolated onto the reef bathymetry. Depth contours, scale and orientation are the same for all subfigures. (**A**,**B**) The inorganic δ^13^C*in* and organic δ^13^C*org* carbon component relative to Vienna Peedee belemnite. (**C**) δ^18^O relative to SMOW. (**D**) Benthic sample depth vs isotopic component values from **A**–**C**. The δ^13^C*in,* δ^13^C*org* and δ^18^O components are shown by the black, blue and green markers respectively.

The patterns of *H. tuna* isotopic composition are complex (Fig. 4) and not constrained to or explained only by location across isobaths. The inorganic/organic δ^13^C and δ^18^O values for *Halimeda tuna* varied both within and across depth gradients on spatial scales of 10–20 m. The inorganic oxygen isotope δ^18^O, from *H. tuna* calcium carbonate sampling (Fig. 4A) generally showed higher values along the fore-reef slope and deeper water (in areas with the greatest benthic water temperature variability) and was lower in water shallower than approximately 20 m (mean and S.D., 27.7 ± 0.92). Similarly, δ^13^C (Fig. 4B) shows complex spatial patterning not following isobaths (mean and S.D., − 18.1 ± 1.6). The lowest values (less than approximately − 19) were typically found deeper on the fore-reef (with an anomalously low, potential outlier, along the NE edge of the 18 m depth contour). The inorganic δ^13^C (Fig. 4C) shows a complex spatial distribution across all depths with highs and lows in some places apparently constrained by the topography, but in other locations not following benthic contours (mean and S.D., 1.6 ± 1.8). The lack of any strong correlation between sample depth and isotopic composition without considering the 3D bathymetry is highlighted in the scatter and lack of any trends of shown between the δ^13^C in, δ^13^C org and δ^18^O components and sample depth shown in Fig. 4D. As shown in Fig. 4D, there were no strong trends between the isotopic composition with depth, and no statistically significant relationships with depth were indicated when examining the isotopic variation in 3 m depth increments, ANOVA (F(4,29) = 3.73, p = 0.49)., ANOVA (F(4,29) = 2.71, p = 0.31), ANOVA (F(4,29) = 1.87, p = 0.30) for the inorganic/organic δ^13^C and δ^18^O signatures respectively.

### δ^18^O estimates of seawater temperature

**Figure 5:**
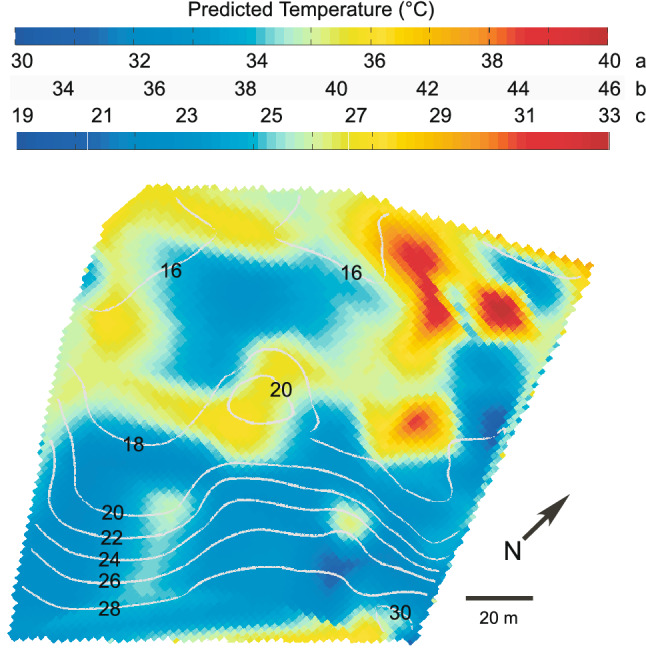
Predicted paleotemperature mapped onto reef bathymetry. Predicted temperatures using isotopic thermometer models, which are variations of the Epstein paleotemperature equation^24,25^ interpolated over the reef bathymetry using the sampled δ^18^O data. The numerals on the grey contour lines on the map indicate depth in m. Because the models are linear, the colour bar gradient and range is identical for all model results, however the absolute scaling varies. The two temperature colour bars above the map are identical. The predicted temperature scales are calculated from: (**A**) late Holocene *Halimeda* from the northwest Caribbean^34^ using Grossman and Ku^64^; (**B)** Miocene *Halimeda* reef material^21^ modified from Böhm et al.^67^; (**C**) *Porities* sp. aragonite collected from the Galapagos^81^.

Using the measured δ^18^O values it was possible to calculate estimates of seawater temperature as predicted by multiple paleothermometer models (Fig. 5) and then interpolate the results onto the reef bathymetry. These models are linear variations of the classic Epstein et al. paleothermometer model^24,25^ : T = a – b(δ^18^O_carb _− δ18O_sw_) + c(δ^18^O_carb _− δ^18^O_sw_)^2^ where T is the predicted temperature, δ^18^O carb is the δ^18^O signature of the carbonate, δ^18^O_sw_ is the δ^18^O signature of the seawater in which the carbonate precipitated, and a, b, and c are constants particular to the specific carbonate-forming organisms sampled and the environment in which they were living. The constants used were from temperature calibrations for late Holocene *Halimeda* samples from the Caribbean Virgin Islands^34^ using the model of Grossman and Ku^64^. Miocene *Halimeda* reef samples^21^ as well as aragonite collected from the coral *Porities* sp. collected from the Galapagos^69^. The predicted results in Fig. 5 suggest seawater temperatures at the time of carbonate formation from about 17 to 46 °C, spanning but greatly exceeding both the yearly minimum and maximum temperatures recorded for Conch Reef. The general predicted temperature patterns do approximate the measured temperature patterns (Fig. 3), however there is temperature heterogeneity within isobaths over a scale of 10′s of m somewhat similar to the patterns indicated in the horizontal temperature anomaly (Fig. 3D).

### Isotope map similarity and temperature

The Numerical Fuzzy Kappa (NFK) measure of map similarity between the predicted temperature and the measured temperature maps, indicated the closest fit (i.e. the closest map similarity with the greatest Kappa value), NFK = 0.61, between δ^18^O predicted temperature (Fig. 5) and the measured mean benthic temperature (Fig. 3A), with decreasing similarity to the benthic temp minimum, NFK = 0.59, the temperature variance, NFK = 0.52, and the mapping of the benthic temperature maximum, NFK = 0.51. It is very difficult to quantitatively compare the patterning of environmental variables between spatial maps, however, the NFK statistic provided a formal mechanism for comparison that did not rely on a qualitative visual assessment^65^. The NFK statistic varies between 0 and 1 with greater values indicating a higher degree of similarity between different maps (1 being identical). For the δ^13^C spatial distribution (Fig. 4B), the map similarity was greatest with the temperature maximum, NFK = 0.66, and benthic temperature variance, NFK = 0.63. The spatial map similarities between measured (i.e. δ^18^O) and predicted metrics do not show as close a fit with the raw bathymetry (Fig. 1B) with NFK values between 0.3 and 0.45. The complex relationship between the benthic topography at Conch Reef and its influence on the local hydrography which varies from minute to seasonal timescales is explored in Leichter et al.^52^. The topographically constrained water flow across the reef front generates high and low temperature anomalies (Fig. 3D) which influence the local benthic environment and complicate the interpretation of spatially and temporally averaged data sets.

## Discussion

The high degree of physical and biological variation within isobaths suggests that simple interpretations of apparent depth effects on biological parameters may be problematic when considering ancient or contemporary systems where extensive sampling within depths is often absent due to logistic or experimental design limitations. This variation, on spatial scales from cm to many meters and likely resulting from processes that vary on temporal scales from minutes to seasons can be of significant ecological importance to benthic sessile organisms, including benthic algae like *H. tuna,* that can be sensitive to relatively small changes in the physical environment. On coral reefs, temperature variability is intrinsically important because of the physiological stresses on the sessile benthic community occurring at both low and high temperatures^68,69^ and also because it can often be inversely correlated with the concentration of dissolved nutrients (like on Conch Reef), which is critical for the growth of autotrophs. Similar physical forcing has been identified in other coral reef habitats^70–73^ and in a wide variety of other shallow water marine environments^74–77^. In the case of Conch Reef, mapping seawater temperature and thermal anomalies onto the reef topography reveals distinct locations on the reef with persistent cooling and warming while spatial mapping of *H. tuna* isotopic signatures also reveals both large and small scale heterogeneity in biogeochemical patterns.

The complex flow of tidal currents and incident internal waves interacts with reef topography that then channels and guides gravity-driven density flows and produces heterogeneity in cool water exposure and residence times that the benthic community experiences^52^. For example, temperature anomalies evident in Fig. 3 are associated with the highs and lows of reef topography that channel the movement of nutrient rich, cooler, and therefore denser waters. The importance of exposure to offshore nutrient sources to benthic primary producers like *Halimeda* is supported by recent experimental evidence by other researchers. Smith et al.^78^ and Vroom et al.^3^ measured higher growth rates of *H. tuna* on the reef slope at Conch Reef with increasing depth. In response to a series of nutrient addition experiments across depths at Conch Reef^78^, Smith et al. found that *H. tuna* at 21 m showed no change in growth rate, but showed an increased growth response at 7 m depth suggesting that the shallower individuals live in nutrient-poor conditions. The heterogenous spatial patterning in *H. tuna* δ^13^C (Fig. 4) that can be related to the efficiency of photosynthetic pathways and physiological fractionation^32,39–41^ are then not surprising as even *H. tuna* growing in relatively close proximity may experience very different growth regimes and phytophysiology due to variation in light intensity, nutrient supply and interspecific interactions with benthic neighbours^45^.

In a paleoecological study, Holmes^34^, analysing the δ^18^O signature from *Halimeda* fragments deposited in sediment from Florida and the Caribbean (Virgin Islands), found evidence of an approximately 4 °C cooler time period roughly 4,000 years before the present in the Northeast Caribbean. Holmes applied the Grossman and Ku^64^ paleothermometer model originally formulated for δ^18^O in benthic foraminifera aragonite whose precipitation is considered to be in close equilibrium to seawater^64,79,80^. The *Halimeda* fragments from the Virgin Islands had a δ^18^O range from − 2.5 to − 2.0 ‰ (mean 2.15 ‰), yielding predicted temperatures from 26.7 to 28.9 °C which, when assuming the seawater δ^18^O to be close to 0 ‰, was within the range of measured values for the area. Holmes^34^ also reported *Halimeda* δ^18^O values of − 3.3 to − 3.0 ‰ for Marquesas Keys in Florida, corresponding to predicted temperatures of 29.8–31.0 °C, also within the range of measured temperature at that location.

On Conch Reef, by contrast, *H. tuna* δ^18^O ranged from − 5.06 to − 2.07 with mean − 3.36 ‰. Following Holmes^34^ and using the equation of Grossman and Ku^64^ and a seawater δ^18^O of + 1.0 estimated for this region from the Global Seawater Database^63^ (similar to the water samples collected during this study), yields predicted temperatures ranging from 30.0 to 41.4 °C with mean of 34.9 °C (Fig. 5). However, these mean temperatures are significantly higher than measured temperatures at the site even during the warmest summer months and at the shallowest depths. Other paleothermometer models based on isotopic signatures from foraminifera, and models from derived from Miocene *Halimeda* and vermitid reefs in Brachert et al.^21^ using equations modified from Böhm et al.^67^, overestimate contemporary reef temperatures on Conch Reef by 10 °C. Whereas, it is interesting to note that an estimate of benthic seawater temperature from a model using contemporary isotopic signatures from *Porities* sp. Aragonite^40,41,66^ yield an estimated temperature range that is similar to the yearly benthic temperature range at Conch reef (see Fig. 2). In this case, the model was derived from coral rather than algal carbonate, and predicts a temperature span from about 19 to 32 °C, and was calibrated on aragonite collected from the Galapagos (see also^81^) at a reef location which undergoes similar yearly temperature fluctuations of 6 to 8 °C and similar cold water upwelling as found along reefs in the Florida Keys.

**Figure 6:**
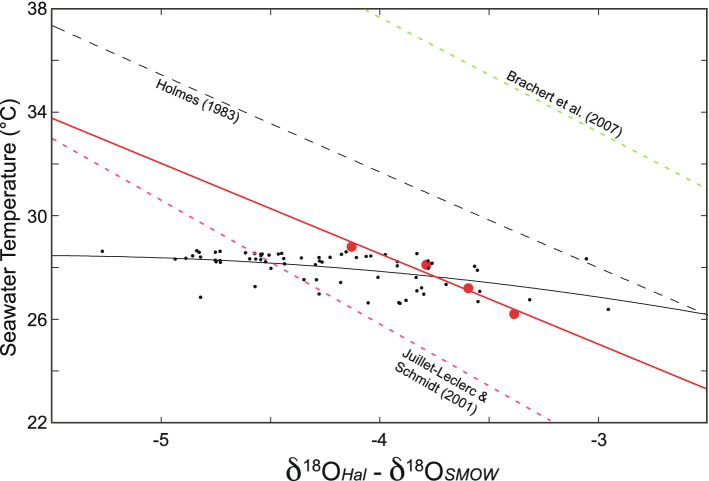
Seawater temperature predictions. Predicted seawater temperature (°C) vs *Halimeda* δ^18^O using mean benthic temperature data from Conch Reef collected during the summer 2004 Benthic Oceanographic Array deployment (black dots, see Fig. 3) and using the mean temperature data collected from 1997–2006 (red dots, see Fig. 2) and binned into 5 m depth intervals from 10 to 35 m (see also^55^). The black line indicates seawater temperature predicted by the best fit equation T (in °C) = 21.0−2.6 (δ^18^O_*Halimeda*_—δ^18^O_seawater_)  − 0.24 (δ^18^O_*Halimeda*_ − δ^18^Os_seawater_)^2^ for the BOA data (r^2^ = 0.26). The red line indicates seawater temperature predicted by the best fit equation T (in °C) = 14.2–3.6 (δ^18^O_*Halimeda*_ − δ^18^O_seawater_) for the binned mean temperature data from 1997–2006 (r^2^ = 0.92). Seawater temperatures predicted by Holmes^34^, Brachert et al.^21^ and Juillet-Leclerc and Schmidt^66^ (see also Fig. 5 above) are shown by the dashed black, green and magenta lines respectively.

The large seasonal and daily temperature fluctuations present at Conch Reef, and the heterogeneous physical conditions experienced by the benthos, even along isobaths, (Fig. 3 and^50^) are imbedded in the time-averaged ^18^O calcification signature sampled from *H. tuna* which has a lifespan anywhere from 1 to > 12 months at Conch Reef^3^ and maximal growth during summer months coincident with the period of maximum temperature variation. Using the spatially sampled temperature field and δ^18^O signatures sampled here (i.e. Fig. 5) it is possible to produce a temperature calibration specific to *Halimeda* and which is likely to be applicable to reefs of the Florida Keys which experience similar hydrography^55^. As shown in Fig. 6, δ^18^O ranged between approximately − 5 and − 2.5 from the sampled locations which experienced a mean temperature of between approximately 26 and 28 °C regardless of depth and in spite of the large range of internal wave induced temperature variation. The best fit model line (solid black line) calculated using a least squares regression suggests a temperature calibration T (in °C) = 21.0−2.6 (δ^18^O_*Halimeda*_ − δ^18^O_seawater_) − 0.24 (δ^18^O_*Halimeda*_ − δ^18^O_seawater_)^2^ with r^2^ = 0.26. It should be noted that this calibration uses the temperature data collected only during the summer months. A better representation of the general temperature field may be calculated from the long time series (1997–2006, from Fig. 2) as the mean benthic temperature binned into 5 m depth intervals (from 10 to 35 m at the Conch Reef site) and shown by the red filled circles. Using these points, suggests a simple linear temperature calibration, T (in °C) = 14.2−3.6 (δ^18^O_*Halimeda*_ − δ^18^O_seawater_) for the binned mean temperature data from 1997–2006 (r^2^ = 0.92) which may be useful for other temperature reconstructions. The overestimates produced by the Holmes^34^ and Brachert et al.^21^ *Halimeda* calibrations and the similarity to the *Porities* aragonite calibration from the upwelling Galapagos reefs is also shown.

An additional way to interpret disparity in the predicted temperature data could be that the measured δ^18^O values in the *H. tuna* aragonite may be lower (less enriched in ^18^O) than would be expected based on estimates of the average seawater δ^18^O for the Florida Straits and known temperatures at the site. If we assume a lower value of seawater δ^18^O, − 1.5 ‰, rather than + 1.0 approximated from^63^ we attain a more reasonable range of predicted temperatures, 22.8–33.8 °C with mean of 27.5 °C. Under the modelling assumptions, the relatively low (less enriched) δ^18^O values we find for *H. tuna* aragonite imply that seawater δ^18^O is approximately 2.5‰ lower than expected based on observed values for the offshore waters of the Florida Keys. Fresh water input is a possible source for low seawater δ^18^O values^27^. Thus, the relatively positive δ^18^O signatures for *H. tuna* at Conch Reef may be indicative of freshwater input into this system, perhaps via frequent heavy rainfall and potentially from inshore or submarine groundwater sources with more negative levels of δ^18^O^88,89^. However, groundwater input has not been identified in prior tracer experiments^82–85^, benthic temperature and salinity data^53,54^, or radium isotopic measurements^86^ and the effects of well-mixed rainfall at the reef front would be negligible. Salinity can vary along with temperature during the arrival of internal waves, however salinity remains in the range of full ocean salinity (e.g. 35 to 36.5 PSU at the site), and does not indicate significant impact of low salinity water at the depths of the reef surface^57^ (and J. Leichter unpublished data). Although there were indications of lower δ^18^O seawater values in offshore water collected at or below 100 m, (0.6 vs 1.0), this small difference does not have a large influence in predicated temperatures, and usually, the water that advects onto the reef front is often well mixed and from much shallower depths^52–57^.

Additionally, although it is of minimal importance in the contemporary *Halimeda* samples collected here, in a paleoenvironmental context, it is also important to consider potential syndepositional and diagenetic effects on *Halimeda* fragments in the sediment. These effects can be significant and under some conditions can occur even within 500 days of burial^30^. The coupled dissolution and re-precipitation of carbonate can lead to a shift in isotopic signature towards equilibrium with the sediment pore waters and away from that of the seawater from which it originally precipitated. The possible isotopic shift towards pore water equilibrium and the complexities in biologically-mediated fractionation during fragment formation suggests that co-occurring inorganically precipitated marine cements within paleocarbonate samples, may provide fidelity for oceanographic geochemical conditions than do the skeletal fragments bound within^87^ .

Wefer and Berger^38^, summarized most cogently, “Vital effects must not be seen merely as a vexation in attempting to reconstruct the environment, however. The task is to extract some of the wealth of ecologic information (illumination, food supply and life history) which is hidden in the disequilibrium patterns of the isotopic signals.” In the case of *Halimeda*, its rapid growth, robust aragonitic form and distinctive fragmentation products create a carbonate sediment amenable to paleoenvironmental reconstruction, with certain caveats. Evidence suggests that the *Halimeda* δ^18^O signature is precipitated in equilibrium with seawater, hence its use as paleothermometer, but, as evidenced here, both δ^18^O and δ^13^C values can show high variability over short spatial scales and within a single depth isobath. A linear model fit to the δ^18^O data, even if sampled along a single depth isobath, may not be easy to interpret, and a mixed or homogenized sample that incorporates the full range of variability underestimates the heterogeneity that may be a real and important component of a dynamic environment. Paleocarbonate samples that show large heterogeneity in both δ^18^O and δ^13^C within a single sedimentary horizon may be indicative of reef settings that have a variable thermal, salinity, and nutrient regime associated with internal waves and tides like at Conch reef.

## Methods

### Study site

The data were collected as part of a series of studies at Conch Reef in the Florida Keys, USA (24°57.0 N, 80°27.3 W), with the National Undersea Research Center, Fig. 1A,B. This location is similar to other reefs in the upper Florida Keys, consisting of a living benthic assemblage encrusting a thin Holocene veneer of scleractinian corals, sponges hydrozoans, and coralline, filamentous and fleshy algae covering a Pleistocene carbonate platform^89–90^. The shallow fore reef at approximately 8–10 m depth and the fore reef both slope gradually (ca. 2–5% slope) to approximately 16 m depth where the slope becomes steeper (ca. 8–15% slope) down to the base of the reef at 30–32 m depth. In some locations, a series of 1–2 m height parallel coral spurs separated by sand channels transect the fore reef slope. At the base of the fore reef, a gradual (1–5%) sand slope extends seaward of the reef for 20–30 km into the deeper channel of the Straits of Florida. At the Conch Reef study site currents are typically alongshore, 0.1–0.5 ms^−1^ flowing towards the northeast with tidal reversals to the southwest^52,57^. Flows are stronger (0.5–2.0 ms^−1^) offshore of the reef tract and driven primarily to the northeast by the Florida Current^54,55^. The surface tides at Conch Reef are mixed semidiurnal with mean amplitude of approximately 0.5–0.75 m^52,57^.

### Environmental sampling

Systematic benthic sampling of the macroalgae *Halimeda tuna* was conducted in conjunction with temperature and current recording instrumentation deployment during a saturation diving project at the NOAA Aquarius habitat. For the high temporal frequency component of the study, the primary apparatus deployed was an extensive temperature-sensor array capable of synchronized, high precision, autonomous sampling for extended periods, known as the Benthic Oceanographic Array (BOA^91,92^). The system consisted of 100 temperature sensors arranged in 10 arrays of 10 elements spaced serially along cables at 15 m intervals. The BOA system allows synchronous sampling of all sensors at a 5-s interval with a resolution and accuracy of 0.007° and 0.04 °C, respectively. Sensor nodes were anchored to the reef by attaching them to 25 cm spikes driven into the bottom or tied onto dead coral substrate. The 10 array cables were connected to a submersible junction box at the BOA control and power supply located at the base of the reef slope at approximately 33 m depth, adjacent to an acoustic Doppler current profiler (ADCP) (RDI Workhorse 600 kHz) with pressure sensor. The ADCP sampled at 1.33 Hz and stored 1-min averages in 1 m vertical bins. Detailed ADCP data are presented in Leichter et al.^57^. An additional BOA strand was fixed to a vertical mooring near the BOA junction box in order to provide water column temperature measurements in approximately 2.5 m intervals between 3 and 30 m depth.

In addition to the high-frequency sampling described above, benthic temperature was measured continuously from 1992 to 2010 at 10, 20, 30 m depth with SBE39 (Seabird Electronics) temperature recorders with 0.001 °C resolution. Data were sampled at 2 to 10 min intervals and averaged in 10 min bins for the long-term data set.

The entire BOA sensor array and the reef topography was mapped in situ using compass board, calibrated measuring tape, and digital depth gauge (see Fig. 1). This procedure located the node positions within less than 0.5 m in horizontal space and to within approximately 0.3 m in depth. The node locations were surveyed relative to fixed markers on the seafloor of known GPS position.

### Isotopic sampling

Mixed samples of *H. tuna*, consisting of entire plants and their segments attached to the holdfasts (excluding loose or dead segments), were hand collected by divers at each node position of the BOA array, rinsed in de-ionized water, and then dried and prepared for isotopic analysis. *H. tuna* is common at this Conch Reef location and samples could usually be collected within 0.5 m of a node. The *H. tuna* samples were divided into two equal parts, half treated with 0.1% HCl to remove calcium carbonate and the remaining half treated with 0.1% NaOH to remove the organic component. The treated samples were then powdered with mortar and pestle and weighed subsamples were placed in tin capsules for isotopic analysis.

Each algal subsample (1–5 mg) of organic material was analyzed for δ^13^C at the Isotrace laboratory of University of Otago by combustion in a Carlo Erba NC2500 elemental analyzer to reduce the samples to CO_2_ and N_2_. The isotopic ratio of CO_2_ and N_2_ were measured by a Europa Scientific 20–20 ANCA Mass Spectrometer operating in continuous flow mode. Delta values were reported against the international standards Vienna Pee Dee Belemnite (VPDB) and atmospheric nitrogen (AIR) for *δ*^13^C and *δ*^15^N respectively. Normalization was applied with a 3-point calibration from 2 glutamic acid international reference materials and a laboratory EDTA standard (Elemental Microanalysis) for carbon (USGS-40 =  − 26.2‰, USGS-41 = 37.8 ‰, EDTA = − 38.93 ‰) and nitrogen (USGS-40 =  − 4.52‰, USGS-41 = 47.57 ‰, EDTA = -0.73 ‰). Results were expressed in standard delta notation where, for example, δ^13^C = [Rsample/Rstd) − 1]*1000 where Rsample = ^13^C/^12^C and Rstd = ^13^C/^12^C of Peedee belemnite limestone. Analytical precision was quantified by comparing results with quality control standards EDTA-OAS and IAEA MA-A-1 (Copepod). All measured values for the quality control standards were in the range of accepted values.

Samples of calcium carbonate from *H. tuna* were independently analysed for δ^18^O. Aliquots (2–3 mg) of ground samples were reacted with 103% H_3_PO_4_ under vacuum overnight. The carbon dioxide produced by this reaction was purified and analysed on a Thermo Advantage isotope ratio mass spectrometer (IRMS) in continuous-flow mode for carbon and oxygen ratios. The same procedure was followed for NBS (National Bureau of Standards) 18 and NBS 19 carbonate standards. Results are reported in the standard delta notation against the standard mean ocean water (SMOW). Consensus values for laboratory standards were obtained from internal laboratory calibration records against primary reference materials, VSMOW, GISP and SLAP (Isotrace – University of Otago).

Seawater samples were collected from the reef slope around the BOA array using diver operated 5L Nisken bottles and from deeper waters offshore of the study site during CTD casts from a small vessel (see also^53,54^). Nisken samples (n = 3–15) were collected at approximately 3, 10, 30, 55, 80, 100 m depth. *δ*^18^O from the samples was determined by the method of equilibration^93^ with CO_2_. Half of a millilitre of a subsample was equilibrated with 12 ml of 0.3% CO_2_ in helium on a Thermo (Bremen) GasBench preparation unit for 18 h at 25.0 ± 0.1 °C. The equilibrated gas was measured in 10 replicates with a Thermo Advantage isotope ratio mass spectrometer (IRMS) in continuous-flow mode and values more than 1 standard deviation from the average were excluded. The filtered average was corrected to the international VSMOW-SLAP isotope scale using a three-point calibration provided by three laboratory standards analysed before and after every batch of 84 samples. In addition, a control sample was measured at every 12th position to correct for instrumental drift. Consensus values for the laboratory standards have been obtained from 6-year internal laboratory calibration records against primary reference materials, VSMOW, GISP and SLAP, external 6-member interlaboratory comparison exercise and by back-calculation from the ~ 170 member IAEA interlaboratory comparison exercise, WICO2012. The laboratory standards and their consensus values are as follows: ICE (*δ*^18^OVSMOW = − 32.097 ± 0.075‰), TAP (*δ*^18^OVSMOW = − 11.432 ± 0.038‰), SEA (*δ*^18^OVSMOW = − 0.029 ± 0.036‰).

### Benthic oceanographic array data analysis

The detailed algorithms used to analyse the BOA sensor array data and the error estimates for the calculated temperature anomalies, can be found in^91^ and for a previous deployment on Conch Reef, FL, in^52^. In general, to calculate the temperature anomaly, for every time step in the data record the mean profile of temperature as a function of depth is calculated with a 3 m vertical averaging length scale. Subtracting this mean profile from the raw data record yields a within depth, horizontal temperature anomaly. Because the thermal field at Conch Reef during the summer months is dominated by vertical gradients, isolating this temperature component allows a detailed examination of the smaller, time-varying component contained in a temperature anomaly and indicative of horizontal gradients across the reef slope. Variation in the temperature anomaly arises from a number of factors including the nature of the cool water incursions on the reef, driven by internal waves, and their interaction with the reef bathymetry. Any within-depth heterogeneity of temperature along isobaths results in nonzero values of the temperature anomaly. To visualize the dataset, values of the temperature and temperature anomaly and the isotopic metrics from all the nodes were interpolated as contour surfaces on a 3-m grid and mapped onto the measured reef bathymetry.

### Map analysis

It is difficult to analyse statistically 2 or 3-dimensional spatial patterns inherent within and between different maps of environmental variables and the topography. However, there is a growing suite of analytical techniques that can quantitatively compare 3-dimensional spatial maps as well as describe map spatial patterns and heterogeneity^65^. The task of quantifying similarity between 3-dimensional maps is complex and the techniques used are only briefly summarized below and the full details can be found in the appropriate geospatial analysis literature^94–99^.

In order to perform similarity comparisons between the spatial maps of benthic temperature and *H. tuna* isotope signatures, the original data maps (i.e. Figs. 3, 4, 5) were first rasterized onto an equivalent 100 × 100 cell grid and the data within each cell normalized such that each value was between 0 and 1. All computations were performed using MATLAB and the Riks Map Comparison tool Kit^99,102^. Map similarity comparisons were based on the Numerical Fuzzy Kappa (NFK) measure which produces a statistic that represents the difference in average similarity between different spatial maps^96–99^. All computations are contingent on the reasonable a priori assumption that the map data are spatially autocorrelated^100,101^. NFK is an extension of the original Kappa algorithm and is based on a misclassification matrix between map cells^65^ but in addition, uses the weighted inputs of neighbouring cells to compute the Kappa statistic based on fuzzy set theory. By doing so, the measure of cell similarity is continuous across the map based on the value and distance of neighbouring cells (in this case with a Gaussian weighting in a 12 cell neighbourhood). The final NFK statistic is able to quantify the level of map difference and can model a human estimate of map ‘similarity’ more closely than the original Kappa^65,96–98,101,103^. The computed NFK statistic varies between 0 and 1 with greater values indicating a higher degree of similarity between different maps (1 being identical).

## Data Availability

Raw data will be made available from the corresponding author following reasonable requests and raw data is also available at SEANOE 10.17882/61,374.

## References

[1] Bathurst RGC (1971). Carbonate sediments and their diagenesis.

[2] Beach K (2003). Variability in the ecophysiology of *Halimeda* spp. (Chlorphyta, Bryopsidales) on Conch Reef, Florida Keys, USA. J. Phycol..

[3] Vroom PS (2003). Field biology of Halimeda tuna (Bryopsidales, Chlorophyta) across a depth gradient: Comparative growth, survivorship, recruitment, and reproduction. Hydrobiologia.

[4] Littler MM, Littler DS, Blair SM, Norris JN (1985). Deepest known plant life discovered on an uncharted seamount. Science.

[5] Littler MM, Littler DS, Blair SM, Norris JN (1986). Deep-water plant communities from an uncharted seamount off San Salvador Island, Bahamas: Distribution, abundance, and primary productivity. Deep Sea Res. Part A Oceanogr. Res. Pap..

[6] Blair SM, Norris JN (1988). The deep-water species of Halimeda Lamouroux (Halimedaceae, Chlorophyta) from San Salvador Island, Bahamas: Species composition, distribution and depth records. Coral Reefs.

[7] Drew EA, Abel KM (1988). Studies on Halimeda: II. Reproduction, particularly the seasonality of gametangia formation, in a number of species from the Great Barrier Reef Province. Coral Reefs.

[8] Johns HD, Moore CH (1988). Reef to basin sediment transport using Halimeda as a sediment tracer, Grand Cayman Island. West Indies. Coral Reefs.

[9] Schupp PJ, Paul VJ (1993). Calcium carbonate and secondary metabolites in tropical seaweeds: Variable effects on herbivorous fishes. Ecology.

[10] Littler MM, Littler DS (1999). Blade abandonment/proliferation: a novel mechanism for rapid epiphyte control in marine macrophytes. Ecology.

[11] Wefer G (1980). Carbonate production by algae Halimeda, Penicillus and Padina. Nature.

[12] Wiman SK, McKendree WG (1975). Distribution of *Halimeda* plants and sediments on and around a patch reef near Old Rhodes Key Florida. J. Sediment. Res..

[13] Mankiewicz C (1988). Occurrence and paleocologic significance of Halimeda in late Miocene reefs, southeastern Spain. Coral Reefs.

[14] Hillis LW (2001). The calcareous reef alga Halimeda (Chlorophyta, Byropsidales): A cretaceous genus that diversified in the cenozoic. Palaeogeogr. Palaeoclimatol. Palaeoecol..

[15] Stanley SM (2008). Effects of global seawater chemistry on biomineralization: Past, present, and future. Chem. Rev..

[16] Wefer G, Berger WH (1981). Stable isotope composition of benthic calcareous algae from Bermuda. J. Sediment. Res..

[17] Flügel E (1988). *Halimeda*: paleontological record and palaeoenvironmental significance. Coral Reefs.

[18] Rao VP (1994). Late quaternary *Halimeda* bioherms and aragonitic faecal pellet-dominated sediments on the carbonate platform of the western continental shelf of India. Mar. Geol..

[19] Stanley SM, Ries JB, Hardie LA (2002). Nonlinear partial differential equations and applications: From the Cover: Low-magnesium calcite produced by coralline algae in seawater of Late Cretaceous composition. Proc. Natl. Acad. Sci..

[20] Stanley SM (2006). Influence of seawater chemistry on biomineralization throughout phanerozoic time: Paleontological and experimental evidence. Palaeogeogr. Palaeoclimatol. Palaeoecol..

[21] Brachert TC (2007). High salinity variability during the early Messinian revealed by stable isotope signatures from vermetid and *Halimeda* reefs of the Mediterranean region. Geol. Romana.

[22] Urey HC (1947). The thermodynamic properties of isotopic substances. J. Chem. Soc..

[23] Urey HC, Lowenstam HA, Epstein S, McKINNEY CR (1951). Measurement of paleotemperatures and temperatures of the Upper Cretaceous of England, Denmark, and the southeastern United States. Geol. Soc. Am. Bull..

[24] Epstein S, Buchsbaum R, Lowenstam H, Urey HC (1951). Carbonate-water isotopic temperature scale. Geol. Soc. Am. Bull..

[25] Epstein S, Buchsbaum R, Lowenstam HA, Urey HC (1953). Revised carbonate-water isotopic temperature scale. Geol. Soc. Am. Bull..

[26] Tabouret H (2010). Simultaneous use of strontium: calcium and barium: calcium ratios in otoliths as markers of habitat: Application to the European eel (*Anguilla anguilla*) in the Adour basin South West France. Mar. Environ. Res..

[27] Ren L (2003). Deconvolving the δ18O seawater component from subseasonal coral δ18O and Sr/Ca at Rarotonga in the southwestern subtropical Pacific for the period 1726 to 1997. Geochim. Cosmochim. Acta.

[28] Grossman EL, Ku T-L (1986). Oxygen and carbon isotope fractionation in biogenic aragonite: Temperature effects. Chem. Geol..

[29] Weber JN, Woodhead PMJ (1972). Temperature dependence of oxygen-18 concentration in reef coral carbonates. J. Geophys. Res..

[30] Patterson WP, Walter LM (1994). Syndepositional diagenesis of modern platform carbonates: Evidence from isotopic and minor element data. Geology.

[31] Hillis-Colinvaux L (1980). Ecology and taxonomy of *Halimeda*: primary producer of coral reefs. Advances in Marine Biology.

[32] Lee D, Carpenter SJ (2001). Isotopic disequilibrium in marine calcareous algae. Chem. Geol..

[33] Wizemann A, Meyer FW, Westphal H (2014). A new model for the calcification of the green macro-alga Halimeda opuntia (Lamouroux). Coral Reefs.

[34] Holmes CW (1983). delta 18 O variations in the Halimeda of Virgin Islands sands; evidence of cool water in the Northeast Caribbean, late Holocene. J. Sediment. Res..

[35] Peach KE, Koch MS, Blackwelder PL, Guerrero-Given D, Kamasawa N (2017). Primary utricle structure of six Halimeda species and potential relevance for ocean acidification tolerance. Bot. Mar..

[36] Peach KE, Koch MS, Blackwelder PL, Manfrino C (2017). Calcification and photophysiology responses to elevated pCO2 in six Halimeda species from contrasting irradiance environments on Little Cayman Island reefs. J. Exp. Mar. Biol. Ecol..

[37] Aharon P (1991). Recorders of reef environment histories: Stable isotopes in corals, giant clams, and calcareous algae. Coral Reefs.

[38] Wefer G, Berger WH (1991). Isotope paleontology: growth and composition of extant calcareous species. Mar. Geol..

[39] Wefer G, Killingley JS (1986). Carbon isotopes in organic matter from a benthic alga Halimeda incrassata (Bermuda): Effects of light intensity. Chem. Geol..

[40] McConnaughey T (1989). 13C and 18O isotopic disequilibrium in biological carbonates: I. Patterns. Geochim. Cosmochim. Acta.

[41] Mcconnaughey T (1989). 13C and 18O isotopic disequilibrium in biological carbonates: II. In vitro simulation of kinetic isotope effects. Geochim. Cosmochim. Acta.

[42] Robbins LL, Knorr PO, Hallock P (2009). Response of Halimeda to ocean acidification: field and laboratory evidence. Biogeosci. Discuss..

[43] Price N, Hamilton S, Tootell J, Smith J (2011). Species-specific consequences of ocean acidification for the calcareous tropical green algae Halimeda. Mar. Ecol. Prog. Ser..

[44] Sinutok S, Hill R, Doblin MA, Wuhrer R, Ralph PJ (2011). Warmer more acidic conditions cause decreased productivity and calcification in subtropical coral reef sediment-dwelling calcifiers. Limnol. Oceanogr..

[45] Barry SC, Frazer TK, Jacoby CA (2013). Production and carbonate dynamics of Halimeda incrassata (Ellis) Lamouroux altered by Thalassia testudinum Banks and Soland ex König. J. Exp. Mar. Biol. Ecol..

[46] Vogel N (2015). Calcareous green alga *Halimeda* tolerates ocean acidification conditions at tropical carbon dioxide seeps: *Halimeda* growing at CO _2_ seeps. Limnol. Oceanogr..

[47] Campbell JE, Fisch J, Langdon C, Paul VJ (2016). Increased temperature mitigates the effects of ocean acidification in calcified green algae (Halimeda spp). Coral Reefs.

[48] Peach K, Koch M, Blackwelder P (2016). Effects of elevated pCO2 and irradiance on growth, photosynthesis and calcification in Halimeda discoidea. Mar. Ecol. Prog. Ser..

[49] Prathep A, Kaewsrikhaw R, Mayakun J, Darakrai A (2018). The effects of light intensity and temperature on the calcification rate of Halimeda macroloba. J. Appl. Phycol..

[50] Teichberg M, Fricke A, Bischof K (2013). Increased physiological performance of the calcifying green macroalga Halimeda opuntia in response to experimental nutrient enrichment on a Caribbean coral reef. Aquat. Bot..

[51] Duarte CM (2018). Stable Isotope (δ13C, δ15N, δ18O, δD) Composition and Nutrient Concentration of Red Sea Primary Producers. Front. Mar. Sci..

[52] Leichter JJ, Deane GB, Stokes MD (2005). Spatial and temporal variability of internal wave forcing on a coral reef. J. Phys. Oceanogr..

[53] Leichter JJ, Stewart HL, Miller SL (2003). Episodic nutrient transport to Florida coral reefs. Limnol. Oceanogr..

[54] Leichter JJ (2007). Nitrogen and oxygen isotopic signatures of subsurface nitrate seaward of the Florida Keys reef tract. Limnol. Oceanogr..

[55] Leichter JJ, Stokes MD, Vilchis LI, Fiechter J (2014). Regional synchrony of temperature variation and internal wave forcing along the Florida Keys reef tract. J. Geophys. Res. Oceans.

[56] Davis KA, Leichter JJ, Hench JL, Monismith SG (2008). Effects of western boundary current dynamics on the internal wave field of the Southeast Florida shelf. J. Geophys. Res..

[57] Leichter JJ, Wing SR, Miller SL, Denny MW (1996). Pulsed delivery of subthermocline water to Conch Reef (Florida Keys) by internal tidal bores. Limnol. Oceanogr..

[58] Lee TN (1992). Influence of Florida current, gyres and wind-driven circulation on transport of larvae and recruitment in the Florida Keys coral reefs. Cont. Shelf Res..

[59] Lee TN, Schott FA, Zantopp R (1985). Florida current: Low-frequency variability as observed with moored current meters during April 1982 to June 1983. Science.

[60] Lee TN, Mayer DA (1987). Low-frequency current variability and spin-off eddies along the shelf off southeast Florida. J. Mar. Res..

[61] Boyer JN, Jones RD, Porter JW, Porter KG (2002). A view from the bridge: external and internal forces affecting the ambient water quality of the Florida Keys National Marine Sanctuary (FKNMS). The Everglades, Florida Bay, and Coral Reefs of the Florida Keys: An Ecosystem Sourcebook.

[62] Kruczynski WL, McManus F, Porter JW, Porter KG (2002). Water quality concerns in the Florida Keys: Sources effects, and solutions. The Everglades, Florida Bay, and Coral Reefs of the Florida Keys: An Ecosystem Sourcebook.

[63] Schmidt, G.A. Global seawater oxygen-18 database. *NASA GISS*http://data.giss.nasa.gov/o18data/ (1999).

[64] Grossman EL, Ku TL (1981). Aragonite-water isotopic paleotemperature scale based on the benthic foraminifer *Hoeglundina elegans*. Geol. Soc. Am. Abstr. Prog..

[65] Rose KA, Roth BM, Smith EP (2009). Skill assessment of spatial maps for oceanographic modeling. J. Mar. Syst..

[66] Juillet-Leclerc A, Schmidt G (2001). A calibration of the oxygen isotope paleothermometer of coral aragonite from *porites*. Geophys. Res. Lett..

[67] Böhm F (2000). Oxygen isotope fractionation in marine aragonite of coralline sponges. Geochim. Cosmochim. Acta.

[68] Brown BE (1997). Coral bleaching: causes and consequences. Coral Reefs.

[69] Knowlton N, Jackson JBC, Bertness MD, Gaines SD, Hay ME (2001). The ecology of coral reefs. Marine Community Ecology.

[70] Wolanski E, Pickard G (1983). Upwelling by internal tides and kelvin waves at the continental shelf break on the Great Barrier Reef. Mar. Freshw. Res..

[71] Wolanski E, Hamner WM (1988). Topographically controlled fronts in the ocean and their biological influence. Science.

[72] Wolanski E, Delesalle B (1995). Upwelling by internal waves, Tahiti, French Polynesia. Cont. Shelf Res..

[73] Wolanski E, Deleersnijder E (1998). Island-generated internal waves at Scott Reef Western Australia. Continent. Shelf Res..

[74] Sandstrom H, Elliott JA (1984). Internal tide and solitons on the Scotian Shelf: A nutrient pump at work. J. Geophys. Res..

[75] Holloway PE (1987). Internal hydraulic jumps and solitons at a shelf break region on the Australian North West Shelf. J. Geophys. Res..

[76] Pineda J (1991). Predictable upwelling and the shoreward transport of planktonic larvae by internal tidal bores. Science.

[77] MacKinnon JA, Gregg MC (2003). Mixing on the late-summer New England shelf—Solibores, shear, and stratification. J. Phys. Oceanogr..

[78] Smith JE, Smith CM, Vroom PS, Beach KL, Miller S (2004). Nutrient and growth dynamics of *Halimeda tuna* on Conch Reef, Florida Keys: Possible influence of internal tides on nutrient status and physiology. Limnol. Oceanogr..

[79] Bemis BE, Spero HJ, Bijma J, Lea DW (1998). Reevaluation of the oxygen isotopic composition of planktonic foraminifera: Experimental results and revised paleotemperature equations. Paleoceanography.

[80] Bemis BE, Spero HJ, Thunell RC (2002). Using species-specific paleotemperature equations with foraminifera: A case study in the Southern California Bight. Mar. Micropaleontol..

[81] Wellington GM, Dunbar RB, Merlen G (1996). Calibration of stable oxygen isotope signatures in Galápagos corals. Paleoceanography.

[82] Lloyd RM (1964). Variations in the oxygen and carbon isotope ratios of florida bay mollusks and their environmental significance. J. Geol..

[83] Swart PK (1996). The stable oxygen and carbon isotopic record from a coral growing in Florida Bay: A 160 year record of climatic and anthropogenic influence. Palaeogeogr. Palaeoclimatol. Palaeoecol..

[84] Corbett DR, Dillon K, Burnett W, Chanton J (2000). Estimating the groundwater contribution into Florida Bay via natural tracers, ^222^ Rn and CH _4_. Limnol. Oceanogr.

[85] Dillon K (2003). Groundwater flow and phosphate dynamics surrounding a high discharge wastewater disposal well in the Florida Keys. J. Hydrol..

[86] Paytan A (2006). Submarine groundwater discharge: An important source of new inorganic nitrogen to coral reef ecosystems. Limnol. Oceanogr..

[87] Gonzalez LA, Lohmann KC (1985). Carbon and oxygen isotopic composition of Holocene reefal carbonates. Geology.

[88] Shinn E (1963). Spur and groove formation on the Florida reef tract. J. Sediment. Res..

[89] Multer HG, Gischler E, Lundberg J, Simmons KR, Shinn EA (2002). Key Largo limestone revisited: Pleistocene shelf-edge facies, Florida Keys, USA. Facies.

[90] Lidz BH, Reich CD, Peterson RL, Shinn EA (2006). New maps, new information: Coral reefs of the Florida keys. J. Coastal Res..

[91] Deane GB, Stokes MD (2002). A robust single-cable sensor array for oceanographic use. IEEE J. Oceanic Eng..

[92] Stokes MD, Leichter JJ, Wing S, Frew R (2011). Temperature variability and algal isotopic heterogeneity on a Floridian coral reef. Mar. Ecol..

[93] Epstein S, Mayeda T (1953). Variation of O18 content of waters from natural sources. Geochim. Cosmochim. Acta.

[94] Monserud RA, Leemans R (1992). Comparing global vegetation maps with the Kappa statistic. Ecol. Model..

[95] Pontius R (2000). Quantification error versus location error in comparison of categorical maps. Photogr. Eng. Remote Sens..

[96] Hagen A (2003). Fuzzy set approach to assessing similarity of categorical maps. Int. J. Geogr. Inf. Sci..

[97] Hagen-Zanker A, Straatman B, Uljee I (2005). Further developments of a fuzzy set map comparison approach. Int. J. Geogr. Inf. Sci..

[98] Hagen-Zanker A (2006). Map comparison methods that simultaneously address overlap and structure. J. Geograph. Syst..

[99] Visser H, de Nijs T (2006). The map comparison kit. Environ. Modell. Softw..

[100] Legendre P, Fortin MJ (1989). Spatial pattern and ecological analysis. Vegetatio.

[101] Fernandez M (2009). Locality uncertainty and the differential performance of four common niche-based modelling techniques. Biodiv. Inf..

[102] MATLAB. version 9.4.0 (R2018a). Natick, Massachusetts: The MathWorks Inc. (2018).

[103] Fernandez M (2009). Locality uncertainty and the differential performance of four common niche-based modeling techniques. Biodiv. Inf..

